# Toward a Computational Neuropsychology of Cognitive Flexibility

**DOI:** 10.3390/brainsci10121000

**Published:** 2020-12-17

**Authors:** Alexander Steinke, Bruno Kopp

**Affiliations:** Department of Neurology, Hannover Medical School, Carl-Neuberg-Straße 1, 30625 Hannover, Germany; kopp.bruno@mh-hannover.de

**Keywords:** cognitive flexibility, Wisconsin Card Sorting Test, computational modeling, Parkinson’s disease, amyotrophic lateral sclerosis

## Abstract

Cognitive inflexibility is a well-documented, yet non-specific corollary of many neurological diseases. Computational modeling of covert cognitive processes supporting cognitive flexibility may provide progress toward nosologically specific aspects of cognitive inflexibility. We review computational models of the Wisconsin Card Sorting Test (WCST), which represents a gold standard for the clinical assessment of cognitive flexibility. A parallel reinforcement-learning (RL) model provides the best conceptualization of individual trial-by-trial WCST responses among all models considered. Clinical applications of the parallel RL model suggest that patients with Parkinson’s disease (PD) and patients with amyotrophic lateral sclerosis (ALS) share a non-specific covert cognitive symptom: bradyphrenia. Impaired stimulus-response learning appears to occur specifically in patients with PD, whereas haphazard responding seems to occur specifically in patients with ALS. Computational modeling hence possesses the potential to reveal nosologically specific profiles of covert cognitive symptoms, which remain undetectable by traditionally applied behavioral methods. The present review exemplifies how computational neuropsychology may advance the assessment of cognitive flexibility. We discuss implications for neuropsychological assessment and directions for future research.

## 1. The Neuropsychology of Cognitive Flexibility

Maintaining goal-directed behavior in the face of novel situations is a fundamental requirement for everyday life. The processes that enable individuals to maintain goal-directedness are subsumed under the term executive control (also called executive function or cognitive control) [1,2,3,4,5,6]. Impaired executive control is a well-documented corollary of various neurological diseases as well as an important predictor of disease progression [7,8,9,10,11,12]. Hence, a major aim of contemporary neuropsychological research is to achieve a better understanding of executive control.

The present review focuses on a particular facet of executive control: cognitive flexibility [4,13,14,15]. Cognitive flexibility refers to the ability to adjust behavior to novel situational demands, rules or priorities in an adaptive manner [4,15,16,17]. There are various standardized neuropsychological assessment tools for cognitive flexibility. These include, for example, the Trail Making Test Part B [18,19,20], the intra/extradimensional attentional set-shifting task [21], and the Wisconsin Card Sorting Test (WCST) [22,23,24]. The WCST is probably the most frequently used tool for the neuropsychological assessment of cognitive flexibility [25].

**Figure 1:**
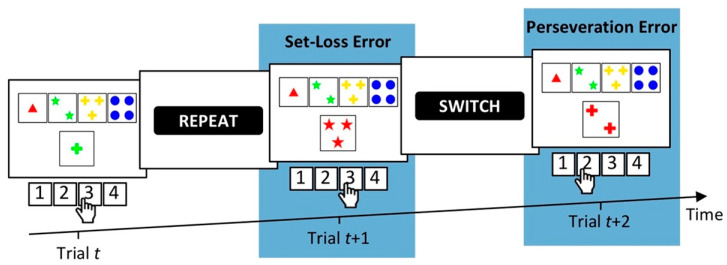
Three consecutive trials on a computerized variant of the Wisconsin Card Sorting Test (cWCST) [11,27,28,29,30]. The stimulus card on Trial *t* depicts one green cross. Applicable categories are the number category (far left key card, response 1), the color category (inner left key card, response 2), and the shape category (inner right key card, response 3). The execution of response 3 indicates the application of the shape category. A succeeding positive feedback cue (i.e., “REPEAT”) indicates that response 3 was correct and that the shape category should be repeated on the upcoming trials. Yet, on Trial *t* + 1, the execution of response 3 indicates the application of the number category. Set-loss errors refer to such erroneous switches of the applied category following positive feedback. A subsequent negative feedback cue (i.e., “SWITCH”) indicates that response 3 was incorrect. Hence, the applied category should be switched. However, on Trial *t* + 2, the execution of response 2 indicates an erroneous repetition of the number category. Perseveration errors refer to such erroneous category repetitions after negative feedback.

The WCST requires participants to sort stimulus cards to key cards according to categories that change periodically. In order to identify the prevailing category, participants need to adjust card sorting to the examiner’s positive and negative feedback, which follows any card sort. Negative feedback indicates that the previously applied category was incorrect, and, accordingly, that participants should switch the applied category. Positive feedback indicates that the previously applied category was correct, and that participants should repeat the applied category. Perseveration errors (PEs) and set-loss errors (SLEs) represent failures to adjust card sorting to these task demands. PEs refer to erroneous category repetitions following negative feedback, and SLEs refer to erroneous category switches following positive feedback. A typical interpretation of increased PE and/or SLE propensities on the WCST is that the assessed participant shows cognitive inflexibility [11]. WCST error propensities usually refer to conditional PE and/or SLE probabilities, e.g., [26] (e.g., conditional PE probabilities equal the number of committed PEs divided by the number of trials following negative feedback). Figure 1 depicts a representative WCST-trial sequence, which illustrates the two types of errors (PE, SLE) that may occur on the WCST.

Beginning with Milner’s [31] seminal work, PE propensities have received the most attention in neuropsychology. Milner [31] investigated the effects of unilateral cortical excisions for the relief of focal epilepsy on PE propensities. Patients with frontal lobe lesions showed massively increased PE propensities when compared to patients with posterior cortical lesions. Two meta-analytical studies confirmed the association between the presence of frontal lobe lesions and increased PE propensities, reporting small (d = −0.32) [32] to large effect sizes (d = −0.97) [33] for elevated PE propensities in patients with frontal lobe lesions when compared to patients with non-frontal brain lesions or healthy controls (HCs) [34]. These meta-analytic findings contributed to the widely held belief that the frontal lobes and related neuroanatomical structures support executive control in general [35,36], and cognitive flexibility in particular [11,32,33].

However, elevated PE propensities not only occur in patients with frontal lobe lesions [37]. For example, Eslinger and Grattan [38] reported increased PE propensities for patients with focal ischemic lesions in the basal ganglia when compared to patients with posterior cortical lesions. Enhanced PE propensities also occur in various neurological patient groups, such as patients with idiopathic Parkinson’s disease (PD) [39], amyotrophic lateral sclerosis (ALS) [29], Alzheimer’s disease [40], Gilles de la Tourette syndrome [41], or primary dystonia [42]. Increased PE propensities also occur in a number of psychiatric patient groups, such as patients with attention deficit hyperactivity disorder [43], eating disorders [44], major depressive disorder [45], or obsessive-compulsive disorder [46]. The ubiquity of increased PE propensities across many neurological diseases and psychiatric disorders suggests that elevated PE propensities may neither be specific neuropsychological symptoms of frontal lobe lesions nor of various clinical conditions [11,47].

The non-specific finding of increased PE propensities across many neurological diseases and psychiatric disorders may result from the impurity of PE propensities [11,13,48,49]. That is, PE propensities may not represent pure correlates of the efficacy of a particular, well-circumscribed cognitive process. Instead, PE propensities may rather reflect the efficacy of a mixture of multiple, yet covert cognitive processes. An impairment of any of these covert cognitive processes could become behaviorally manifest as increased PE propensities [11]. Due to this process impurity, PE propensities may not achieve nosological specificity across a range of neurological diseases and psychiatric disorders. Thus, neuropsychological assessment of cognitive flexibility via PE propensities should probably be considered as a first step, subject to further improvement, rather than as a goal state of affairs. The purpose of the present article is to review recent progress toward computational modeling of covert cognitive processes that may be related to the commitment of overt behavioral errors on the WCST, and to analyze how computational modeling may contribute to the development of next-generation neuropsychological assessment methods.

**Figure 2:**
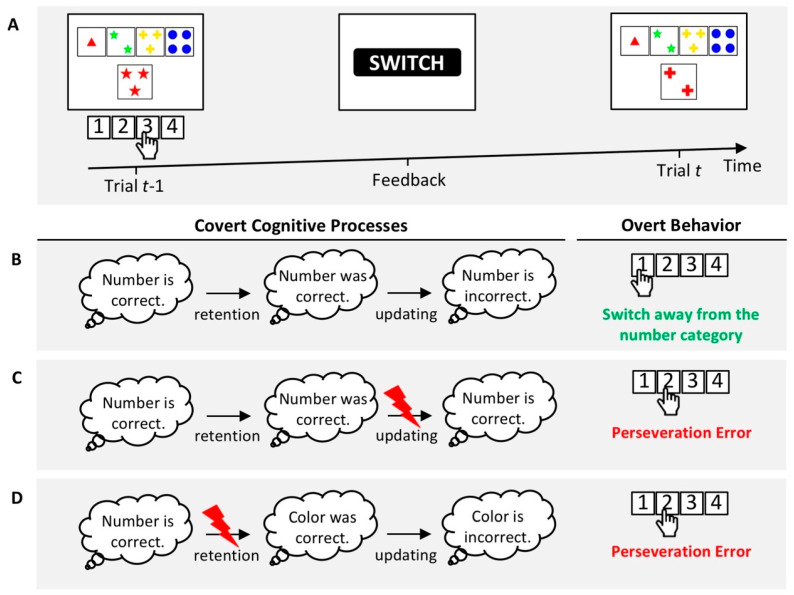
Increased perseveration error (PE) propensities may result from separable impairments of covert cognitive processes. (**A**) An exemplary sequence on a computerized WCST variant. On Trial *t* − 1, the execution of response 3 indicates the application of the number category. A subsequently presented negative feedback cue (i.e., “SWITCH”) indicates that the application of the number category was incorrect. Thus, a switch away from the number category is requested on Trial *t*. (**B**) A successful switch away from the number category on Trial *t* may rely on a number of covert cognitive processes. For example, participants must retain the assumption about the prevailing category on Trial *t* − 1 (i.e., “number is correct”) until they receive a feedback cue (i.e., “number was correct”). Next, participants must update the retained assumption about the prevailing category by received feedback (i.e., “number is incorrect”). At the level of overt behavior on Trial *t*, the execution of response 1 indicates the application of the color category, i.e., a successful switch away from the number category. (**C**) A covert cognitive symptom may describe impaired updating following received feedback. In this example, impaired updating results in the assumption that the number category is still correct, although the received negative feedback indicates that the application of the number category was incorrect. At the level of overt behavior, the execution of response 2 indicates an erroneous repetition of the number category, i.e., a PE. (**D**) Another covert cognitive symptom may describe impaired retention. In this example, impaired retention results in the assumption that the color category was correct. A received negative feedback (i.e., “Color is incorrect”) renders a subsequent application of the number or shape category likely. At the level of overt behavior, the execution of response 2 indicates the application of the number category, i.e., a PE. Please note that we do not wish to imply that these covert cognitive processes are conscious (i.e., the depicted clouds might just as well reflect implicit processes).

Based on the assumption that PE propensities reflect the efficacy of a mixture of covert cognitive processes, similar increased PE propensities across various clinical conditions could arise from (partially) separable impairments of covert cognitive processes. However, such covert cognitive symptoms may not yet be detectable by behavioral WCST measures because any impairment of covert cognitive processes may become behaviorally manifest as elevated PE propensities. Figure 2 presents an illustrative example of this reasoning.

We developed our computational research program in the context of two neurological diseases, i.e., PD and ALS. A loss of dopaminergic neurons in nigro-striatal pathways primarily characterizes PD [50,51]. In contrast, a loss of upper and lower motor neurons in the brain and spinal cord neurons characterizes ALS [52]. There is evidence for increased PE propensities in both patients with PD and patients with ALS [29,39]. Despite this neuropsychological commonality between patients with PD and patients with ALS, the neurodegenerative alterations that occur in patients with PD could affect a set of covert cognitive processes that remain spared in patients with ALS, who, in contrast, show impairments in a distinct set of covert cognitive processes [11]. Thus, while patients with PD and patients with ALS remain indiscernible by analyses of overt PE propensities, these patient groups may nevertheless show (partially) dissociable impairments of covert cognitive processes (i.e., covert cognitive symptoms). The assessment of covert cognitive processes in patients with PD and patients with ALS could provide initial progress toward the detection of nosologically specific aspects of cognitive inflexibility.

## 2. Assessing Covert Cognitive Processes on the WCST

Before we review recent advances with regard to computational modeling of covert cognitive processes on the WCST, we will give an overview of common methodological approaches to covert cognitive processes. Of particular interest for this overview is the utility of the discussed methodological approaches for an individual-based assessment of covert cognitive processes.

### 2.1. Dissociating Patterns of Erroneous Responses

**Figure 3:**
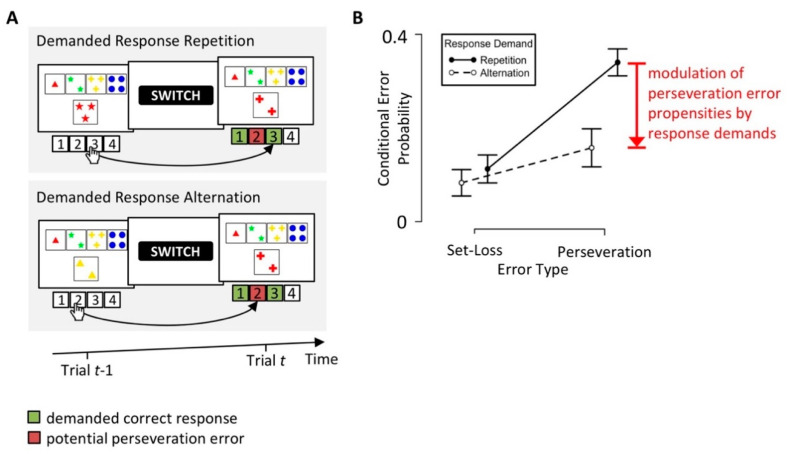
A modulation of PE propensities by response demands. (**A**) In a recent behavioral study [26], we stratified PE by response demands. With a demanded response repetition, the commitment of a PE (i.e., the re-application of the number category by executing response 2 on Trial *t*) implies an alternation of the previously executed response (i.e., response 3 on Trial *t* − 1). With a demanded response alternation, the commitment of a PE (i.e., the re-application of the number category by executing response 2 on Trial *t*) implies the repetition of the previously executed response (i.e., response 2 on Trial *t* − 1). (**B**) We found a modulation of PE propensities by response demands [26]. Participants showed reduced PE propensities on trials with a demanded response alternation when compared to trials with a demanded response repetition. Please note that we did not find evidence for a modulation of set-loss error (SLE) propensities by response demands.

The dissociation of patterns of erroneous responses represents a common approach to the identification and isolation of covert cognitive processes on the WCST [26,28,53,54]. For example, in a recent behavioral study [26] of neurological inpatients who completed a short paper-and-pencil version of the WCST (the modified-WCST; M-WCST) [55], we stratified PE and SLE by response demands (see Figure 3). We found reduced PE propensities with PEs that implied a response repetition (i.e., “Demanded Response Alternation” in Figure 3A), when compared to PEs, which implied a response alternation (i.e., “Demanded Response Repetition” in Figure 3A). These results suggest a modulation of PE propensities by response demands; PEs become less likely when they imply repeating the response that has received a negative feedback on the previous trial. We concluded that participants not only learn to avoid re-applications of categories following a received negative feedback. In addition, participants also learn to avoid re-executions of responses after received negative feedback.

We replicated the modulation of PE propensities by response demands in a large sample of young volunteers (*N* = 375) who completed a computerized WCST (cWCST) variant [56]. This successful replication suggests that response demands modulate PE propensities not only on a paper-and-pencil variant of the WCST (i.e., M-WCST), but also on a computerized WCST variant. The successful replication of the modulation of PE propensities by response demands in a large sample of young volunteers also suggests that neurological inpatients, as well as individuals with no known brain damage, show this behavioral phenomenon.

Analyses of patterns of erroneous responses may allow for the detection of particular behavioral effects on the WCST (e.g., a modulation of PE propensities by response demands), which in turn allow inferences about covert cognitive processes (e.g., learning to avoid re-executions of particular responses following negative feedback). However, analyses of erroneous responses still refer to overt behavioral events, rendering conclusions about actual covert cognitive processes difficult. Thus, the dissociation of patterns of erroneous responses does not represent a satisfactory approach to the assessment of covert cognitive processes.

### 2.2. Identifying and Isolating Latent Variables

Computational methods provide an alternative approach to covert cognitive processes on the WCST [48,57]. Computational methods identify and isolate latent variables (as opposed to observable variables, such as WCST error propensities) from observed behavior. Latent variables reflect the efficacy of covert cognitive processes that may support WCST responding. In contrast to the dissociation of patterns of erroneous responses, computational methods allow for inferences closer to the level of covert cognitive processes.

#### 2.2.1. Factor Analyses

Factor analyses identify sets of latent variables that explain variance common to WCST scores [58,59,60]. Factor-analytical WCST studies consistently revealed a single latent variable, which could indicate a general executive control ability [57]. However, factor-analytical WCST studies remain inconclusive about the number of additionally identifiable latent variables [57]. Furthermore, it remains difficult to infer which covert cognitive processes are actually reflected by these latent variables and how these covert cognitive processes could interact [57]. Thus, factor analyses are of limited utility for the assessment of covert cognitive processes.

#### 2.2.2. Computational Modeling

In contrast to factor analyses, computational models explicitly formalize covert cognitive processes and the way in which these covert cognitive processes interact by mathematical expressions [61,62,63,64,65,66]. Computational models thereby allow one (1) to systematically test hypotheses about covert cognitive processes and (2) to estimate sets of latent variables that reflect the efficacy of the assumed covert cognitive processes [61,62,63,64,67,68].

In the first case, computational models represent hypotheses about covert cognitive processes [68]. Evaluations of competing computational models allow one to test hypotheses about covert cognitive processes. A common method for the evaluation of computational models is to compare their abilities to predict observed behavior [68,69]. The computational model that provides the best prediction of observed behavior may also give the best conceptualization of covert cognitive processes among the compared computational models. Another method for the evaluation of computational models is to compare their abilities to simulate particular behavioral phenomena, such as observed PE and SLE propensities [68]. If a computational model does not simulate all behavioral phenomena of interest, then that computational model should be considered as falsified [68].

There are several computational models for the WCST [48,70,71,72,73,74,75,76,77,78,79,80]. These computational models typically belong to one of two subclasses: neural network models or mechanistic models [48]. Most computational models of the WCST are neural network models (e.g., [71,72]). Neural network models are biologically inspired sets of computational units (referred to as cells or neurons) [81,82]. Interconnections of computational units usually mirror cerebral structures that instantiate specific covert cognitive processes [48,74]. For example, Caso and Cooper [74] proposed a neural network model of the WCST that incorporates cortical and striatal learning mechanisms. “Lesions” (i.e., alterations of latent variables) to computational units that reflect striatal learning mechanisms were considered as a model of pathophysiological changes in patients with PD (see also [83]). The lesioned neural network model produced PE propensities comparable to those observed in a sample of patients with PD [29]. The authors concluded that the proposed neural network model represents a biologically plausible model of (impaired) striatal learning mechanisms in patients with PD.

Neural network models allow the simulation of general patterns of WCST error propensities, such as increased PE propensities as found in patients with PD [74]. However, neural network models incorporate very large numbers of latent variables, rendering their precise estimation for individual participants difficult [48]. In addition, the enormous number of latent variables complicates their psychological interpretation. Thus, neural network models provide limited utility for the assessment of covert cognitive processes.

The second family of computational models of the WCST are so-called mechanistic models [48,67]. In mechanistic models, straightforward computational mechanisms instantiate the assumed covert cognitive processes. Mechanistic models typically incorporate a small number of latent variables, which can be robustly estimated from individual trial-by-trial WCST responses [48,67]. Thus, in contrast to neural network models, mechanistic models provide sets of latent variables for each assessed participant. Moreover, latent variables obtained from mechanistic models—as opposed to latent variables obtained from factor analyses or neural network models—serve as psychologically interpretable metrics for covert cognitive processes. Against this background, mechanistic models could provide a suitable approach to the assessment of covert cognitive processes on the WCST—an approach which we will refer to as computational neuropsychology [48,67,84,85,86,87,88,89].

## 3. Toward a Computational Neuropsychology of Cognitive Flexibility

Computational neuropsychology may provide progress toward nosologically specific aspects of cognitive inflexibility. That is, analyses of latent variables of mechanistic models could reveal disease-specific covert cognitive symptoms of neurological conditions, which yet remain undetectable by traditionally applied behavioral methods.

During the remainder of this article, we aim to elucidate whether computational neuropsychology possesses the potential to reveal nosologically specific profiles of covert cognitive symptoms. We will therefore review and compare mechanistic models of the WCST. Having identified the most suitable mechanistic model of the WCST among all models considered, we will discuss exemplary clinical applications of this mechanistic model in patients with PD and patients with ALS. In order to shed light on the nosological specificity of covert cognitive symptoms, we will compare profiles of covert cognitive symptoms of patients with PD and patients with ALS.

### 3.1. Mechanistic Models of the WCST

#### 3.1.1. The Attentional-Updating Model

The attentional-updating (AU) model by Bishara et al. [48] represents an established mechanistic model of the WCST. Core to the AU model is the assumption that participants form attentional prioritizations (APs) of categories. A high AP of a category results in a high probability of applying that category on a particular trial. APs of categories are trial-wise updated following received feedback. Following a received positive feedback, the AP of the applied category will increase, and AP of not-applied categories will decrease (and vice versa for negative feedback). Thus, following received positive feedback, the repetition of a category becomes more likely, whereas a switch of the applied category becomes more likely after received negative feedback. An attentional focus mechanism modulates the strength of updating of AP: a high AP of a particular category results in strong updating of that AP. In contrast, a low AP of a particular category results in weak updating of that AP.

**Figure 4:**
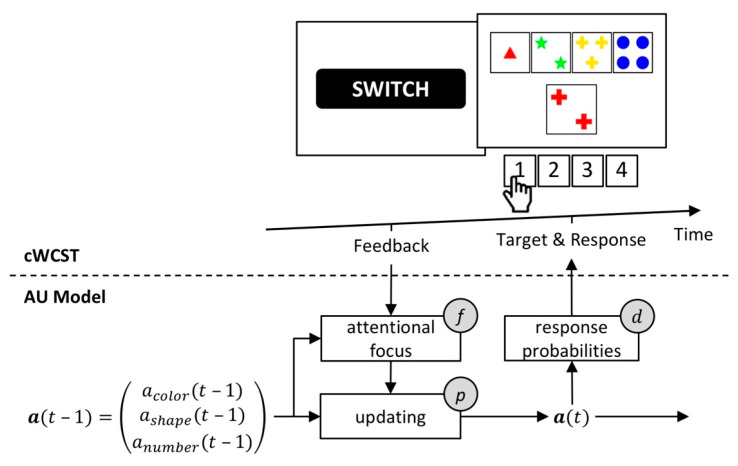
A schematic representation of the attentional-updating (AU) model [48]. Top: an exemplary sequence on a computerized WCST. Bottom: central to the AU model are attentional prioritizations (APs) of categories, **a**(*t*). APs from the previous trial **a**(*t* − 1) are updated in response to a received feedback. Individual sensitivity parameters *p* quantify the overall strengths of updating. There are separate sensitivity parameters for trails following positive and negative feedback (not depicted). An attentional focus mechanism further modulates the strength of updating of AP (i.e., a high AP of a category results in strong updating of that AP and vice versa). An individual attentional focus parameter *f* quantifies the extent to which the magnitude of an AP modulates updating of that AP. An individual response variability parameter *d* quantifies the extent to which response probabilities correspond to updated AP, **a**(*t*).

The AU model incorporates four individual latent variables. Sensitivity parameters quantify the overall strengths of updating of AP following received feedback. The AU model includes separate sensitivity parameters for positive and negative feedback, enabling different individual strengths of updating following positive and negative feedback. An attentional focus parameter quantifies the extent to which magnitudes of AP modulate the strength of updating of AP. A response variability parameter quantifies how well responding corresponds to AP. Figure 4 gives a schematic depiction of the AU model.

The AU model successfully contributed to a number of clinical studies [67,78,90]. For example, Bishara et al. [48] applied the AU model to study covert cognitive symptoms in substance dependent individuals. Substance dependent individuals showed a decreased sensitivity for negative feedback as well as increased response variability when compared to a control group. The AU model also contributed to a lesion mapping study [91]. Results of this lesion mapping study suggest an association between lesions in the right prefrontal cortex (PFC) and the sensitivity parameter for negative feedback. In a model evaluation study, the AU model successfully simulated individual PE and SLE propensities of patients with PD and HC participants who completed a cWCST variant [48,67,91].

#### 3.1.2. The Cognitive Reinforcement-Learning Model

The cognitive reinforcement-learning (RL) model [56] is based on the well-established mathematical framework of reinforcement learning [89,92,93,94,95,96,97,98]. Core to the cognitive RL model is the assumption that participants form feedback predictions for the application of categories. A high feedback prediction indicates a strong prediction of positive feedback for the application of a category. High feedback predictions for a category also relate to a high probability of applying that category. Feedback predictions for categories are trial-wise updated in response to received feedback. Following received positive feedback, feedback predictions for the applied category will increase. After received negative feedback, feedback predictions for the applied category will decrease. Prediction errors modulate the strength of updating of feedback predictions. Prediction errors equal the difference between the received feedback and the predicted feedback. Large prediction errors result in stronger updating of feedback predictions.

The cognitive RL model incorporates two mechanisms that are not inherent parts of canonical RL models [92]. First, a retention mechanism describes the transfer of feedback predictions from one trial to the next [99,100]. Second, a “soft-max” rule gives response probabilities as a function of feedback predictions on a particular trial [92,101,102,103].

**Figure 5:**
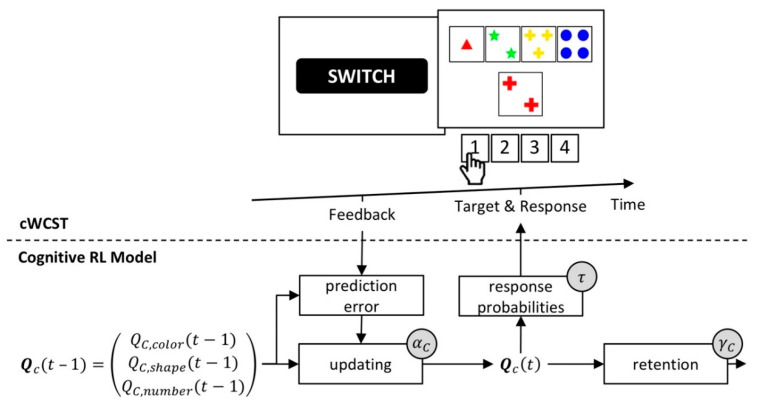
A schematic representation of the cognitive reinforcement-learning (RL) model. Top: an exemplary sequence on a computerized WCST. Bottom: core to the cognitive RL model are feedback predictions for the application of categories, **Q**_c_(*t*). A prediction error updates feedback predictions from the previous trial **Q**_c_(*t* − 1) following received feedback. Individual cognitive learning rates α_c_ quantify the strength of the updating of feedback predictions by prediction errors. There are separate individual cognitive learning rates for received positive and negative feedback (not depicted). A soft-max rule gives response probabilities as a function of updated feedback predictions. The individual inverse temperature parameter τ quantifies how well response probabilities accord to updated feedback predictions. A retention mechanism gives the extent to which feedback predictions transfer to the next trial. The individual cognitive retention rate γ_c_ quantifies the strength of retention of feedback predictions.

The cognitive RL model comprises four individual latent variables. Cognitive learning rates quantify the extent to which prediction errors update feedback predictions. There are separate cognitive learning rates for received positive and negative feedback [89,104,105,106]. A cognitive retention rate quantifies the extent to which feedback predictions transfer from one trial to the next [99,100]. An inverse temperature parameter quantifies how well executed responses correspond to feedback predictions [101,102,103]. Figure 5 gives a schematic depiction of the cognitive RL model.

#### 3.1.3. The Parallel Reinforcement-Learning Model

Based on the finding of a modulation of PE propensities by response demands (see Figure 3), we hypothesized that participants learn at two parallel levels on the WCST [26]. Category-level (putatively cortical) learning implies that participants tend to repeat the applied category on trials following positive feedback, and that they tend to switch the applied category on trials following negative feedback. Participants might also learn at the level of responses. Response-level (putatively striatal) learning implies that participants tend to repeat the execution of a particular response following positive feedback, and that participants tend to avoid the re-execution of a response following negative feedback.

The parallel RL model [56] constitutes a mathematical formalization of category- and response-level learning [26]. Cognitive RL (as in the cognitive RL model) serves as an instantiation of category-level learning. In addition, sensorimotor RL serves as an instantiation of response-level learning. Hence, the parallel RL model constitutes an extended variant of the cognitive RL model (see Figure 5).

Sensorimotor RL is solely concerned with feedback predictions for the execution of responses irrespective of associated categories. A high feedback prediction for the execution of a response results in a high probability of executing that response. Feedback predictions for responses are trial-wise updated following received feedback. Following a received positive feedback, feedback predictions for the executed response will increase, whereas feedback predictions for the executed response will decrease after a received negative feedback. Thus, following a received positive feedback, the repetition of a response execution becomes more likely, whereas a switch of the executed response becomes more likely after a received negative feedback. Prediction errors (i.e., the difference between the received feedback and the predicted feedback for the execution of a particular response) modulate the strength of updating of feedback predictions for responses. Sensorimotor RL also incorporates a retention mechanism that describes the transfer of feedback predictions for responses from one trial to the next [99,100]. The parallel RL model adds feedback predictions for responses to feedback predictions for categories on any trial. A soft-max function gives response probabilities as a function of these integrated feedback predictions [92,101,102,103].

**Figure 6:**
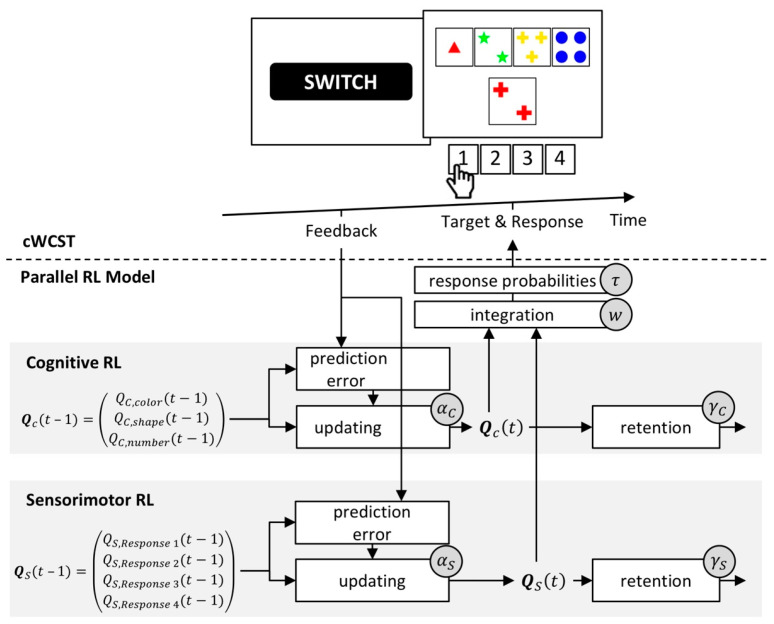
A schematic representation of the parallel RL model. Top: an exemplary sequence on a computerized WCST. Bottom: the parallel RL model incorporates independent cognitive and sensorimotor RL (upper and lower grey bar, respectively). Central to cognitive and sensorimotor RL are feedback predictions for the application of categories **Q**_c_(*t*) and the execution of responses **Q**_s_(*t*), respectively. Cognitive and sensorimotor prediction errors update feedback predictions for categories **Q**_c_(*t* − 1) and responses **Q**_s_(*t* − 1) from the previous trial in response to a received feedback. Individual cognitive α_c_ and sensorimotor learning rates α_s_ quantify the strengths of updating by prediction errors. There are separate learning rates for received positive and negative feedback at cognitive and sensorimotor levels (not depicted). The parallel RL adds feedback predictions for responses to those of categories on any trial. A weighting parameter *w* quantifies the relative strength of cognitive over sensorimotor RL. Response probabilities result from integrated feedback predictions. An inverse temperature parameter τ quantifies how well response probabilities accord to integrated feedback predictions. Cognitive and sensorimotor retention mechanisms ensure that feedback predictions for categories and responses transfer from one trial to the next. Cognitive γ_c_ and sensorimotor retention rates γ_s_ quantify the strengths of retention.

The parallel RL model incorporates eight individual latent variables. Separate cognitive and sensorimotor learning rates quantify the extents to which prediction errors update feedback predictions for categories and responses, respectively. There are separate learning rates for received positive and negative feedback at both cognitive and sensorimotor levels [89,104,105]. Separate retention rates at cognitive and sensorimotor levels [99,100] quantify the extents to which feedback predictions for categories and responses transfer from trial to trial. A weighting parameter quantifies the relative strength of cognitive over sensorimotor RL. An inverse temperature parameter [101,102,103] expresses how well executed responses correspond to integrated feedback predictions. Figure 6 gives a schematic depiction of the parallel RL model.

#### 3.1.4. Comparison of Mechanistic Models

In a recent model comparison study [56], we evaluated the AU model [48], the cognitive RL model, and the parallel RL model on a large sample of healthy volunteers (*N* = 375) who completed a cWCST variant [30].

We evaluated mechanistic models by predictive accuracies [107,108]. Predictive accuracies quantify how well a mechanistic model predicts observed trial-by-trial cWCST responses. The cognitive and the parallel RL model showed better predictive accuracies than the AU model for most participants. These results suggest that RL models provide a better conceptualization of trial-by-trial cWCST responses than the AU model.

RL models differ from the AU model [48] with regard to updating mechanisms. In RL models, prediction errors modulate the strength of the updating of feedback predictions. Prediction errors ensure that updating of feedback predictions is stronger when the correspondence between the received and the predicted feedback is poor. For example, a participant receives positive feedback for the application of a category that had a low feedback prediction (i.e., indicating the prediction of a negative feedback for that category). Thus, the prediction of feedback for this category was poor, resulting in a high prediction error. Hence, updating of feedback prediction for this category will be strong, facilitating the re-application of the category that produced a positive feedback. In the AU model, an attentional focus mechanism ensures that updating of AP of a particular category is less strong when the AP of that category was low. In the example mentioned above, updating of AP will be less strong since the AP of that category was low. Hence, the attentional focus mechanism complicates the re-application of the category that produced a positive feedback. Thus, RL models incorporate more efficient adaptation of card sorting to changing task demands in comparison to the AU model.

RL models further differ from the AU model with regard to retention mechanisms. In RL models, retention mechanisms attenuate feedback predictions from one trial to the next [99,100]. In the AU model, APs transfer from trial-to-trial without attenuation. RL models also differ from the AU model with regard to the computation of response probabilities. A soft-max rule gives response probabilities in RL models [92,101,102,103]. In contrast, an algorithm that divides single AP by the overall sum of AP gives response probabilities in the AU model. Lastly, in RL models, prediction errors update single feedback predictions on any trial (i.e., prediction errors only update feedback predictions for the applied category and/or the executed response). The AU model assumes that all APs of categories are updated on any trial (i.e., after a received positive feedback, the AP of the applied category increases, and all other AP decrease, and vice versa for a received negative feedback).

Our model comparison study [56] remains inconclusive about which particular mechanism of RL models gives a better conceptualization of trial-by-trial cWCST responses than the corresponding AU mechanism. Future studies should explicitly compare the discussed model mechanisms. Such studies could evaluate predictive accuracies of mechanistic models that solely differ with regard to one of the contrasted mechanisms.

Suitable mechanistic models of the WCST should account for a wide range of behavioral phenomena [68]. In our model comparison study [56], the benchmark for all mechanistic models was (1) a successful simulation of individual PE and SLE propensities as well as (2) a successful simulation of the modulation of perseveration propensities by response demands (see Figure 3) [26]. The parallel RL model clearly outperformed the cognitive RL model and the AU model with regard to simulations of these behavioral phenomena. All mechanistic models under consideration simulated individual PE and SLE propensities. However, only the parallel RL model simulated the modulation of PE propensities by response demands.

Against this background, the parallel RL model, which incorporates cognitive and sensorimotor RL as computational instantiations of category- and response-level learning, represents a suitable mechanistic model of the cWCST. In contrast, the cognitive RL model and the state-of-the-art AU model are insufficient mechanistic models of the cWCST.

### 3.2. Assessing Covert Cognitive Symptoms in Neurological Diseases

In order to elucidate whether computational neuropsychology possesses the potential to reveal nosologically specific profiles of covert cognitive symptoms, we will review exemplary applications of the parallel RL model [56] in patients with PD and patients with ALS.

#### 3.2.1. Parkinson’s Disease

In a recent computational study [109], we characterized covert cognitive symptoms associated with PD pathophysiology. Therefore, we reanalyzed data from 16 patients with PD and 34 matched HC participants who completed a cWCST variant [110] by means of the parallel RL model.

**Figure 7:**
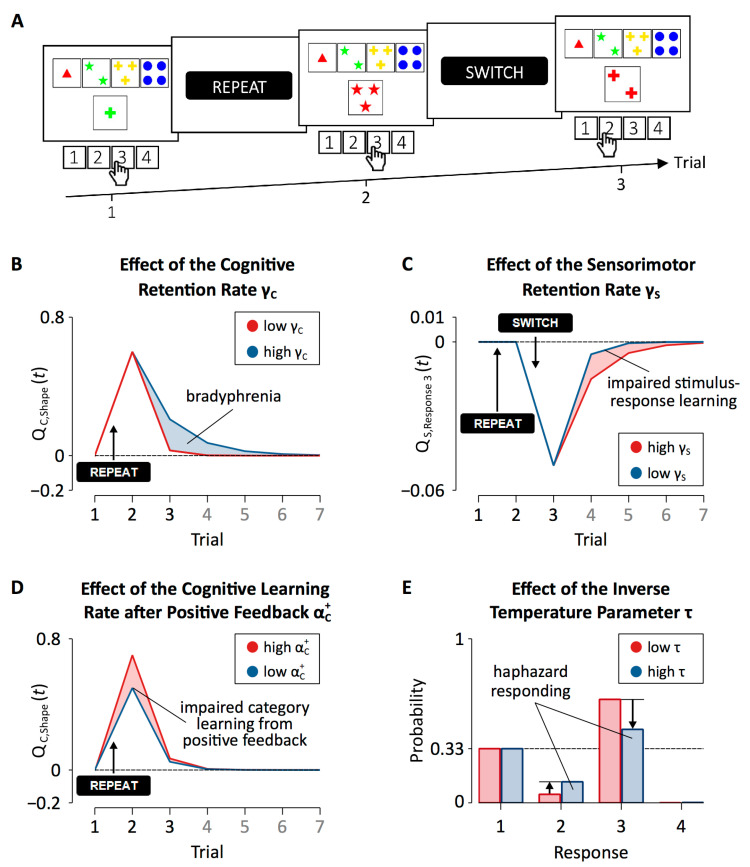
Exemplary effects of between-group variations of latent variables of the parallel RL model. (**A**) A showcase trial sequence on the cWCST as presented in Figure 1. (**B**) Feedback predictions for the application of the shape category across seven trials (panel A shows the first three of them). A positive feedback followed the application of the shape category on Trial 1, which increased feedback predictions for the shape category. With high values of cognitive retention rates (i.e., γ_c_), such as seen in patients with Parkinson’s disease (PD) and patients with amyotrophic lateral sclerosis (ALS), feedback predictions for categories remain at high levels when transferring to the next trial. (**C**) Feedback predictions for the execution of response 3. The execution of response 3 produced a positive feedback on Trial 1. Since sensorimotor learning rates for positive feedback were virtually zero in all studies, there was no updating of feedback predictions for the execution of response 3 following received positive feedback. On Trial 2, the execution of response 3 produced a negative feedback which decreased feedback predictions for response 3. With low sensorimotor retention rates (i.e., γ_s_), such as seen in patients with PD, feedback predictions for the execution of responses retain lower levels of activation from trial-to-trial. (**D**) Feedback predictions for the application of the shape category. With low values of cognitive learning rates for positive feedback (i.e., α_c_^+^), such as seen in patients with PD “on” dopamine (DA) medication, feedback predictions for categories receive reduced levels of activation following received positive feedback. (**E**) Response probabilities on Trial 3. The probability of executing response 3 is the highest (application of the shape category), followed by the probability of executing response 1 (application of the color category) and the probability of executing response 2 (application of the number category). Increased inverse temperature parameters (i.e., τ), such as seen in patients with ALS, attenuate differences between response probabilities. Hence, increased inverse temperature parameters bias response probabilities toward a uniform probability of 0.33. We computed the presented effects of latent variables by varying exclusively the latent variable of interest at arbitrary values while holding all other latent variables constant. Figure 7 is adapted from [109,115].

Patients with PD showed increased cognitive retention rates when compared to HC participants. With high cognitive retention rates, feedback predictions for categories that produced a negative feedback remain at high levels when transferring to the next trial. Hence, the erroneous repetition of such categories becomes more likely (see Figure 7B), rendering category-level learning inflexible. We concluded that increased cognitive retention rates are an expression of bradyphrenia (i.e., “inflexibility of thought”), which represents a hallmark cognitive symptom of PD, at the level of covert cognitive processes [111,112,113,114].

Patients with PD also showed reduced sensorimotor retention rates when compared to HC participants. Reduced sensorimotor retention rates indicate that feedback predictions for responses transfer less strongly from trial to trial (see Figure 7C). Thus, in patients with PD, responding on a particular cWCST-trial is less strongly affected by previous feedback predictions for responses when compared to HC participants. That is, responding of patients with PD appears less repetitive (following positive feedback) or alternating (following negative feedback). The finding of decreased sensorimotor retention rates in patients with PD may correspond to impaired stimulus-response learning (or, with regard to the cWCST, selecting a key card by executing a response), which was repeatedly reported for patients with PD [116,117,118].

#### 3.2.2. Dopamine Replacement Therapy in Patients with PD

In our recent computational study of patients with PD [109], we also characterized covert cognitive symptoms associated with the administration of dopamine (DA) replacement therapy. Therefore, patients with PD were assessed both “on” and “off” DA medication (i.e., after withdrawal of DA medication) [110].

DA replacement therapy aims to alleviate motor symptoms in patients with PD by restoring missing DA in nigro-striatal DA systems. However, adjusting systemic DA replacement solely at the best possible motility may incur cognitive side effects. Optimal DA replacement in the nigro-striatal DA systems may lead to DA overdosing in less affected DA systems, such as the meso-limbic and/or meso-cortical DA systems. Thereby, DA replacement therapy may induce cognitive impairments [12,119,120,121,122,123,124].

The application of the parallel RL model revealed that DA replacement therapy in patients with PD increased cognitive retention rates. Thus, DA replacement therapy seems to induce bradyphrenic side effects (see Figure 7B). DA replacement therapy in patients with PD also reduced cognitive learning rates following positive feedback, indicating that DA replacement therapy in patients with PD induces another covert cognitive symptom: impaired category learning from positive feedback (see Figure 7D).

The meso-cortical DA systems support cognitive flexibility [125,126,127], whereas the meso-limbic DA systems support anticipation of feedback [128,129]. Thus, distinct DA systems could give rise to the reported iatrogenic cognitive impairments induced by DA replacement therapy [130]. An overstimulation of meso-cortical DA systems might cause bradyphrenic side effects, whereas an overstimulation of meso-limbic DA systems might impair category learning from positive feedback [109].

#### 3.2.3. Amyotrophic Lateral Sclerosis

In another computational study [115], we characterized covert cognitive symptoms associated with ALS pathophysiology. Therefore, we reanalyzed data from 18 patients with ALS and 21 matched HC participants who completed a cWCST variant [29] by means of the parallel RL model.

Patients with ALS showed increased cognitive retention rates when compared to HC participants (see Figure 7B). These results suggest that bradyphrenia does not specifically occur in patients with PD. In contrast, bradyphrenia may rather constitute a disease-nonspecific covert cognitive symptom associated with pathophysiological changes in both patients with PD and patients with ALS.

Patients with ALS also showed increased inverse temperature parameters in comparison to HC participants. The inverse temperature parameter expresses how well finally executed responses correspond to integrated feedback predictions for categories and responses [92,101,102,103]. Higher configurations of the inverse temperature parameter indicate that responding is more independent of integrated feedback predictions. Thus, with high inverse temperature parameters, responding appears to be more haphazard (see Figure 7E). These results suggest that ALS pathophysiology comprises another covert cognitive symptom: haphazard responding. Haphazard responding may relate to motor impairments in patients with ALS. For example, haphazard responding could arise from deficient fine motor skills of patients with ALS that obstruct successful cWCST responding [115,131].

#### 3.2.4. Comparison

Traditionally applied behavioral methods for the neuropsychological assessment of cognitive flexibility do not possess sufficient nosological specificity. For example, patients with PD and patients with ALS show increased PE propensities [29,39]. Thus, the finding of increased PE propensities is neither specific to patients with PD nor to those with ALS. We proposed that computational neuropsychology could provide progress with regard to the detection of nosologically specific aspects of cognitive inflexibility.

Our exemplary comparison of profiles of covert cognitive symptoms of patients with PD and patients with ALS corroborates this hypothesis [109,115]. Computational modeling revealed a disease-nonspecific alteration in latent variables. Patients with PD and patients with ALS showed increased cognitive retention rates. These results suggest that bradyphrenia constitutes a disease-nonspecific covert cognitive symptom, which characterizes both patient groups. DA medication in patients with PD further increased cognitive retention rates, indicating that DA medication in patients with PD incurred bradyphrenic side effects.

Computational modeling also revealed PD- and ALS-specific covert cognitive symptoms. Patients with PD, but not those with ALS, showed decreased sensorimotor retention rates when compared to HC participants. Decreased sensorimotor retention rates could indicate impaired stimulus-response learning in patents with PD. DA medication in patients with PD decreased cognitive learning rates after positive feedback. Thus, DA medication in patients with PD could induce impaired category learning from positive feedback. Lastly, only patients with ALS showed increased inverse temperature parameters when compared to HC participants. Increased inverse temperature parameters in patients with ALS may indicate haphazard responding.

**Figure 8:**
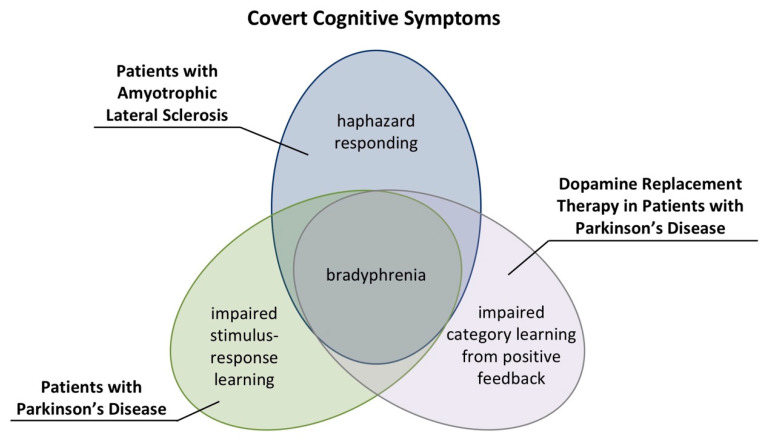
Profiles of covert cognitive symptoms in patients with PD and patients with ALS [109,115].

The reported covert cognitive symptoms in patients with PD and patients with ALS demonstrate that computational neuropsychology possesses the potential to reveal nosologically specific profiles of covert cognitive symptoms. Figure 8 summarizes profiles of covert cognitive symptoms in patients with PD and patients with ALS [109,115].

## 4. Implications for Neuropsychological Assessment

The present review demonstrates how computational neuropsychology may provide progress with regard to the neuropsychological assessment of cognitive flexibility [109,115]. First, as delineated above, computational neuropsychology possesses the potential to reveal nosologically specific profiles of covert cognitive symptoms, which remain yet undetectable by traditional behavioral methods of neuropsychological assessment.

Second, traditional behavioral neuropsychological assessment refers to cognitive assessment, yet the referenced cognitive processes remain unobservable. For example, a typical inference from the presence of enhanced WCST error propensities would be that the assessed participant shows cognitive inflexibility [4,34,49,132]. Hence, behavioral neuropsychological assessment involves drawing inferences that go beyond behavioral observations. In contrast, computational neuropsychology offers a technique for the assessment of latent variables. As latent variables reflect the efficacy of assumed covert cognitive processes, computational neuropsychology may allow for inferences at the level of covert cognitive processes.

Third, behavioral neuropsychological assessment typically refers to vaguely defined cognitive symptoms. That is, cognitive symptoms are often verbal re-descriptions of behavioral observations. For example, Naville [133] observed a lack of voluntary attention, initiative, spontaneous interest, and capacity for effort in patients with encephalitis lethargica, which was also noted in patients with PD [134]. Naville summarized this observation as bradyphrenia [134]. Bradyphrenia literally translates to “slowness of thought”. Hence, a number of studies of bradyphrenia utilized response time tasks [112]. However, prolonged response times, when considered as an expression of bradyphrenia, are likely to be confounded with bradykinesia (i.e., “slowness of movement”) [112,135,136]. Hence, response times are not process pure because they intermingle bradyphrenia and bradykinesia.

Another interpretation of bradyphrenia refers to cognitive akinesia [134], rendering bradyphrenia better conceived as “inflexibility of thought”. Therefore, a number of studies investigated bradyphrenia by means of neuropsychological tests, which target aspects of attentional or cognitive flexibility [137,138]. The example of the bradyphrenia construct illustrates that the reliance on vague semantic definitions renders the interpretation of behavioral studies as indicating particular cognitive symptoms difficult or even impossible.

Computational neuropsychology provides indicators of covert cognitive symptoms along with explicit definitions of their meaning. For example, we considered increased cognitive retention rates as an indicator of bradyphrenia (see above). We showed how increased cognitive retention rates render category-level learning inflexible (see Figure 7B). In the long run, explicit computational definitions may replace the state-of-the-art, yet ambiguous semantic constructs that typically back-bone behavioral neuropsychological assessment.

## 5. Outlook

The ultimate success of computational neuropsychology for neuropsychological assessment depends on further studies of validity and reliability [139]. A common method for the validation of computational models is to assess their ability to simulate particular behavioral phenomena [68,100,140,141]. In our recent model comparison study [56], we assessed mechanistic models with regard to their ability to simulate PE and SLE propensities as well as the modulation of PE propensities by response demands [26]. The AU model [48], as well as the cognitive RL model, failed to simulate the modulation of PE propensities by response demands. In contrast, the parallel RL model successfully simulated this behavioral effect. Thus, the parallel RL model may represent a valid mechanistic model of the cWCST with regard to the studied behavioral phenomena. However, the parallel RL model only remains valid until it fails to explain yet unnoticed behavioral phenomena, or until yet to be specified computational models explain the known behavioral phenomena in a more parsimonious manner [68,140].

Future studies should also validate computational models with regard to their proposed neural underpinnings. For example, cortical brain areas may primarily support cognitive RL, whereas sub-cortical, striatal brain areas may primarily support sensorimotor RL [26]. Confirmatory brain imaging studies should test this hypothesis. Such studies could make use of individual trial-by-trial variables provided by the parallel RL model. For example, individual trial-wise cognitive and sensorimotor prediction errors could correlate with activation patterns in cortical and/or striatal brain areas as revealed by functional magnetic resonance imaging [142,143].

Future studies should also investigate the clinical validity of computational modeling [139,144]. With regard to the parallel RL model, we found increased cognitive retention rates in patients with PD and patients with ALS, which we considered an expression of bradyphrenia. These results suggest an association between increased cognitive retention rates and brain dysfunctions that are common to patients with PD and patients with ALS [115]. Both PD and ALS pathophysiology affect the premotor cortex and the dorsolateral PFC (Broadman areas 4, 6, 8, and 9) [145,146,147]. Hence, increased cognitive retention rates may relate to dysfunctions in these cortical areas. Our finding that patients with PD “on” DA medication showed even more exaggerated cognitive retention rates supports this hypothesis. That is, DA replacement therapy in patients with PD may overstimulate meso-cortical DA systems [119,120,122,123].

Alterations in other latent variables of the parallel RL model could specifically relate to pathophysiological characteristics of patients with PD and patients with ALS [115]. Only patients with PD showed decreased sensorimotor retention rates. Striatal brain areas may primarily support sensorimotor RL [26]. Striatal brain areas are also strongly affected in patients with PD [50,51]. Thus, decreased sensorimotor retention rates could relate to striatal dysfunctions in patients with PD. DA replacement therapy in patients with PD decreased cognitive learning rates following positive feedback. As discussed above, decreased cognitive learning rates could relate to an overstimulation of meso-limbic DA systems induced by DA replacement therapy in patients with PD. Lastly, only patients with ALS showed increased inverse temperature parameters. Thus, increased inverse temperature parameters could possibly relate to motor cortex dysfunctions associated with ALS pathophysiology [52]. Future research should explicitly test these hypothesized relationships between alterations in latent variables and pathophysiological characteristics of patients with PD and patients with ALS [115]. Such studies could combine computational modeling with brain imaging and/or lesion-(covert)-symptom mapping [91,143,148].

Computational models should provide reliable latent variable estimation from observed behavior [139,141]. Parameter recovery allows one to assess the reliability of parameter estimation [139,141]. Parameter recovery studies simulate behavior by a mechanistic model using a pre-defined set of latent variables. If latent variable estimation is reliable, there should be a close correspondence between the pre-defined set of latent variables and latent variables estimated from simulated behavior. An investigation of parameter recovery [56] suggests that a configuration of the parallel RL that incorporates a weighting parameter (see Figure 6) did not provide reliable latent variable estimation. However, a configuration of the parallel RL model that does not incorporate a weighting parameter provided reliable parameter estimation [56]. We utilized this less complex configuration of the parallel RL model (i.e., a configuration without a weighting parameter) to study covert cognitive symptoms in patients with PD and patients with ALS. These results of parameter recovery suggest that reducing model complexity (i.e., the number of latent variables) may improve the reliability of latent variable estimation.

It could also be advisable to assess other facets of reliability of latent variables, such as temporal stability and/or internal consistency [139,149]. Studies addressing latent variables repeatedly over time should investigate the temporal stability of latent variables, as assessed by test–retest reliability [150]. Studies addressing latent variables in other contexts should investigate the internal consistency of latent variables, as assessed by split-half reliability. Split-half reliability methods apply to any assessment tool that can be split into subsets of trials, such as the cWCST [150,151].

The WCST served as an exemplary assessment tool for the present review. However, we would like to highlight that computational neuropsychology is not limited to the WCST. In fact, computational neuropsychology should be applicable to many assessment tools. The sole requirements are (1) that there is a mechanistic model of a participant’s performance, which provides a set of latent variables at the level of individuals, and (2) that these latent variables can be estimated from observed behavior with sufficient precision. The precision of latent variable estimation can be increased with the number of analyzed participants [109,115]. Hence, computational neuropsychology may be particularly suitable for (re-)analyses of large datasets, such as those available from open science approaches [152,153] or multi-lab studies [154].

## 6. Conclusions

Increased PE propensities are a well-documented behavioral finding in many neurological patient groups. This disease-nonspecific finding suggests that cognitive inflexibility constitutes a cognitive symptom common to all these neurological diseases. However, elevated PE propensities may actually arise from shared and disease-specific impairments of covert cognitive processes supporting cognitive flexibility. The present review demonstrates that computational neuropsychology possesses the potential to reveal such nosologically specific profiles of covert cognitive symptoms, which remain undiscoverable through traditional behavioral neuropsychology. We conclude that computational neuropsychology offers a potential route to the advancement of neuropsychological assessment.
